# Supplementary material for: Linking Soil Microbial Functional Profiles to Fungal Disease Resistance in Winter Barley Under Different Fertilisation Regimes
Source: Plants (Basel). 2025 Oct 18;14(20):3199. doi: 10.3390/plants14203199 (PMC12567115; doi:10.3390/plants14203199)
Supplement: Supplementary file 1 [file plants-14-03199-s001.zip › plants-3884874-supplementary.pdf]

Figure S1. Degree of attack, %.

Symptoms of plant desease	
**PLOT 1/ VARIANT 1**	**Degree of attack, %**
1. Powdery mildew of barley (*Blumeria graminis*)	10
2. Net blotch (*Pyrenophora teres)*	33
3. Brown rust (*Puccinia hordei*)	13
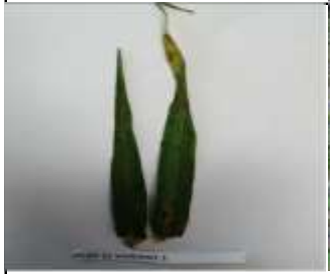 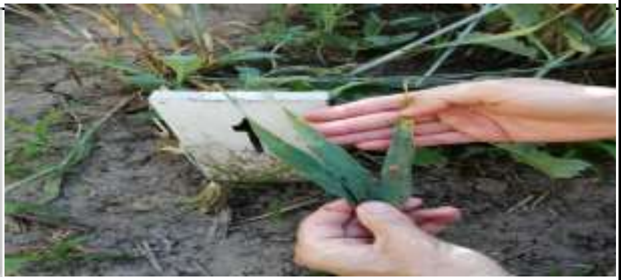	
**PLOT 1/ VARIANT 2**	
1. Powdery mildew of barley (*Blumeria graminis*)	12
2. Net blotch (*Pyrenophora teres)*	33
3. Brown rust (*Puccinia hordei*)	31
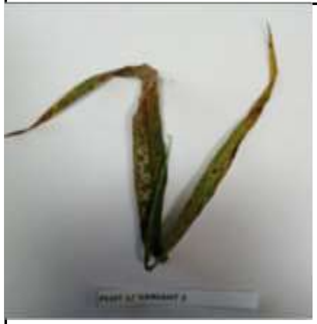 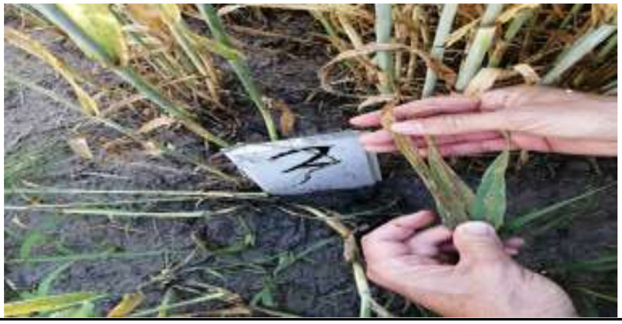	
**PLOT 1/ VARIANT 3**	
1. Powdery mildew of barley (*Blumeria graminis*)	35

2. Net blotch (*Pyrenophora teres)*	25
3. Brown rust (*Puccinia hordei*)	12
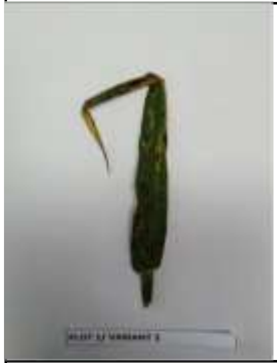 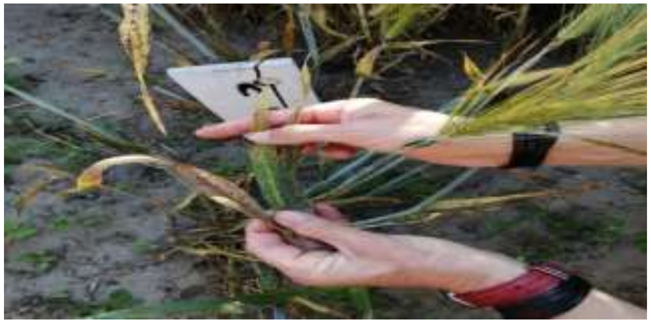	
**PLOT 1/ VARIANT 4**	
1. Powdery mildew of barley (*Blumeria graminis*)	15
2. Net blotch (*Pyrenophora teres)*	38
3. Brown rust (*Puccinia hordei*)	20
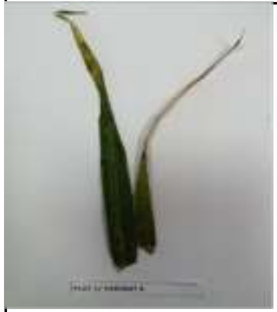 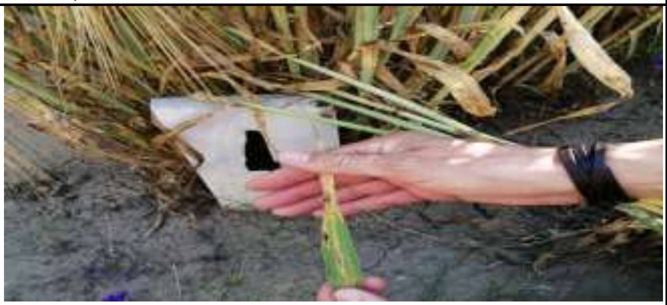	
**PLOT 1/ VARIANT 5**	
1. Powdery mildew of barley (*Blumeria graminis*)	18
2. Net blotch (*Pyrenophora teres)*	11
3. Brown rust (*Puccinia hordei*)	32

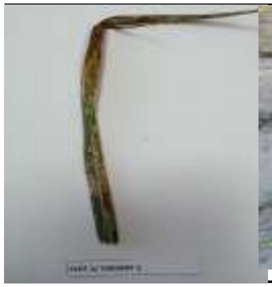

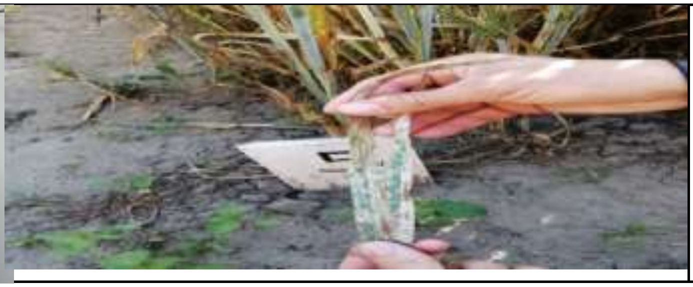

s on barley demonstration plot

PLOT 2/ VARIANT 1	Degree of attack, %
1. Powdery mildew of barley (*Blumeria graminis*)	55
2. Net blotch (*Pyrenophora teres)*	20
3. Brown rust (*Puccinia hordei*)	80
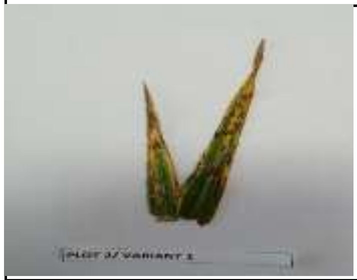 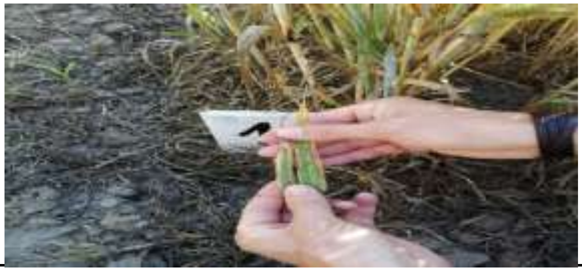	
**PLOT 2/ VARIANT 2**	
1. Powdery mildew of barley (*Blumeria graminis*)	65
2. Net blotch (*Pyrenophora teres)*	20
3. Brown rust (*Puccinia hordei*)	5
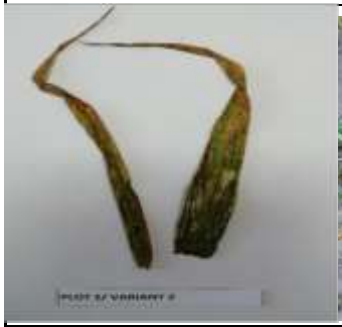 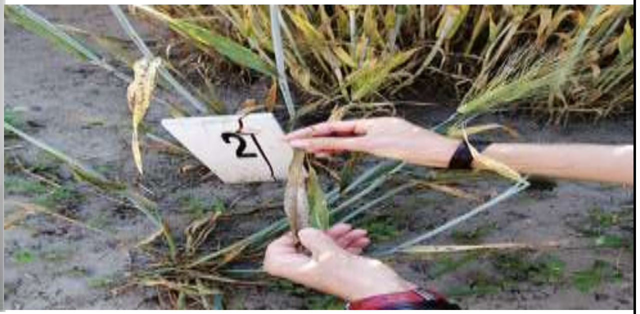	
**PLOT 2/ VARIANT 3**	
1. Powdery mildew of barley (*Blumeria graminis*)	10

2. Net blotch (*Pyrenophora teres)*	70
3. Brown rust (*Puccinia hordei*)	25
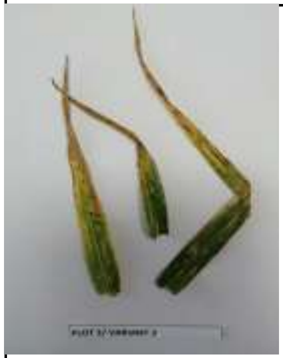 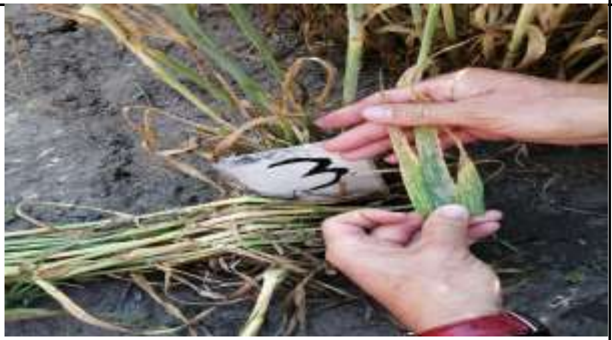	
**PLOT 2/ VARIANT 4**	
1. Powdery mildew of barley (*Blumeria graminis*)	25
2. Net blotch (*Pyrenophora teres)*	30
3. Brown rust (*Puccinia hordei*)	76
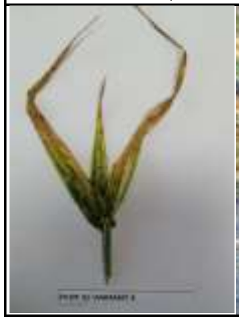 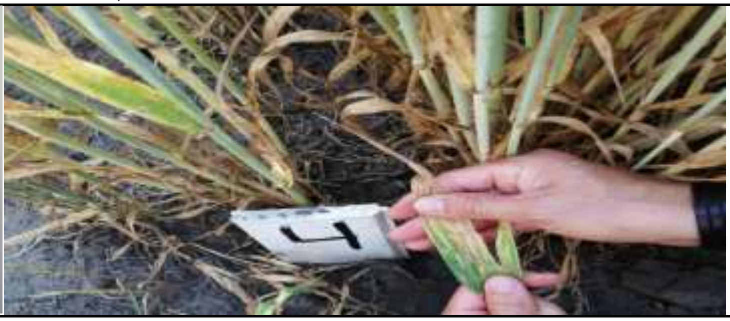	
**PLOT 2/ VARIANT 5**	
1. Powdery mildew of barley (*Blumeria graminis*)	72
2. Net blotch (*Pyrenophora teres)*	25
3. Brown rust (*Puccinia hordei*)	10

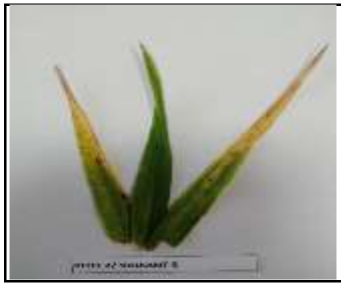

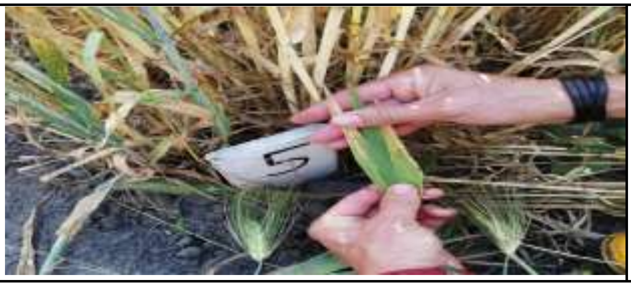
5
